# Species composition of the largest shark fin retail-market in mainland China

**DOI:** 10.1038/s41598-020-69555-1

**Published:** 2020-07-31

**Authors:** Diego Cardeñosa, Andrew T. Fields, Elizabeth A. Babcock, Stanley K. H. Shea, Kevin A. Feldheim, Demian D. Chapman

**Affiliations:** 1 School of Marine and Atmospheric Science, Stony Brook University, Stony Brook, NY 11794 USA; 2 Fundación Colombia Azul, Bogotá, Colombia; 3 Department of Marine Biology and Ecology, Rosenstiel School of Marine and Atmospheric Science, University of Miami, Miami, FL 33149 USA; 4 BLOOM Association, c/o, ADMCF, Suite 2405, Queen’s Road Central, Hong Kong, China; 5 Pritzker Laboratory for Molecular Systematics and Evolution, The Field Museum, Chicago, IL 60605 USA; 6 Department of Biological Sciences, Florida International University, 3000 NE 151st Street, North Miami, FL 33181 USA

**Keywords:** Genetic markers, Sequencing, Genetics, Ocean sciences, Marine biology

## Abstract

Species-specific monitoring through large shark fin market surveys has been a valuable data source to estimate global catches and international shark fin trade dynamics. Hong Kong and Guangzhou, mainland China, are the largest shark fin markets and consumption centers in the world. We used molecular identification protocols on randomly collected processed fin trimmings (n = 2000) and non-parametric species estimators to investigate the species composition of the Guangzhou retail market and compare the species diversity between the Guangzhou and Hong Kong shark fin retail markets. Species diversity was similar between both trade hubs with a small subset of species dominating the composition. The blue shark (*Prionace glauca*) was the most common species overall followed by the CITES-listed silky shark (*Carcharhinus falciformis*), scalloped hammerhead shark (*Sphyrna lewini*), smooth hammerhead shark (*S. zygaena*) and shortfin mako shark (*Isurus oxyrinchus*). Our results support previous indications of high connectivity between the shark fin markets of Hong Kong and mainland China and suggest that systematic studies of other fin trade hubs within Mainland China and stronger law-enforcement protocols and capacity building are needed.

## Introduction

Many shark populations have declined in the last four decades, mainly due to overexploitation to supply the demand for their fins in Asia and meat in many other countries^1–4^. Mainland China was historically the world’s second largest importer of shark fins and foremost consumer of shark fin soup, yet very little is known about the species composition of shark fins in this trade hub^2^. Most global shark catch and trade data are aggregated, unreported, or misidentified at the species level, hampering species-specific management and product traceability throughout supply chains^5,6^. Species-specific monitoring of the shark trade has become a priority for most countries, in part because of international treaty obligations under the Convention on International Trade in Endangered Species of Wild Fauna and Flora (CITES) where several shark species traded in large volumes have been listed on Appendix II^7,8^.

One key source of species-specific information on the international trade of shark fins has been the systematic studies of the dried fin market of Hong Kong Special Administrative Region of the People’s Republic of China (hereafter referred to as Hong Kong)^1,7,9^. Hong Kong is arguably the world’s largest and most consistent importer and re-exporter of shark fins, a small-scale processor (i.e., removing extraneous tissue and preparing fins for the retail market), and a major consumer of shark fin soup^2,10^. However, despite its consistency it is unwise to assume that the species composition of Hong Kong is representative of all of the international fin trade because there are other hubs in Asia, each with their own internal dynamics, supply chains, and customer preferences^2^. The fin trade in Mainland China, for example, differs from Hong Kong in at least two major respects: it is also a shark fin producer through its distant water fishing fleet^2^ and Guangdong province in southern China hosts a substantial fin processing industry, where fins landed or imported into China (including many from Hong Kong) are dried, soaked in water, bleached and trimmed of extraneous tissue (e.g., muscle, skin, cartilage) to isolate the ceratotrichia that are the primary soup ingredient^2,11^. Shark fins in the city of Guangzhou are obtained from processing plants in Guangdong and then sold to local costumers, and restaurants and wholesalers in Beijing, Shanghai and other cities^11^. Interview surveys with local traders in Guangzhou suggested that shark fins in this market include tiger sharks (*Galeocerdo cuvier*), silky sharks (*Carcharhinus falciformis*), blue sharks (*Prionace glauca*) and oceanic whitetip sharks (*C. longimanus*)^11^, although no species-specific survey has ever been conducted in this market, hampering a direct comparison with other shark fin markets.

The objectives of this study were to (i) investigate for the first time the species composition of the Guangzhou dried fin market and (ii) compare the species composition of the Guangzhou and neighboring Hong Kong retail markets in terms of species diversity and most commonly traded species. From 2014 onwards, fin market surveys in Hong Kong have used fin-trimmings, an inexpensively sold byproduct of fin processing that is composed of pieces of fin with cartilage that have been cut away from the ceratotrichia, as an affordable market proxy^7,9^. This same proxy was used in Guangzhou.

## Methods

Guangzhou, the capital city of the Guangdong Province, is the largest shark fin trade hub in mainland China^11^ and lies 129 km from Hong Kong. Its retail market is more centralized than Hong Kong’s, comprising a mall that includes mixed wholesale-retail stalls and shops with serial numbers, where shark fins and other highly-priced traditional Chinese medicine products are sold^11^. The shark fin retail market of Guangzhou was sampled every 2–3 months from June 2015 to August 2017 for a total of ten sampling events. We generated a list of all vendors based on their serial numbers, and ten random vendors were randomly selected from the complete shop list each sampling event. Sampling events consisted of purchasing two bags of processed shark fin trimmings from each randomly selected vendor, yielding a total of 20 bags of trimmings per sampling, similar to the sampling method described by Fields et al.^9^ and Cardeñosa et al.^7^ for Hong Kong.

The contents of each bag were counted, numbered, and ten trimmings were randomly selected for species identification. Genomic DNA was extracted following the protocols used by Refs.^12,13^. Briefly, a small piece of tissue (processed fin trimming) of approximately 2 mm^2^ was cut and placed in a PCR tube with 200 μl of 10% Chelex Resin (BioRad). Processed fin samples were agitated under water before extraction to reduce potential contamination. Once in Chelex, samples were heated at 60 °C for 20 min and then at 99 °C for 25 min, followed by a brief centrifugation. Each 25 μl PCR included 0.5 μl of extracted DNA, 12.5 μl of GoTaq Hot Start Green Master Mix (Promega) and five mini-barcoding primers for the cytochrome oxidase I (COI) locus as described by Ref.^14^. These mini-barcoding primers yield up to three fragments for each template (650 bp, 150 bp, 200 bp) but typically only one or both of the smaller fragments due to low quality gDNA present in these processed fins. The multiplex PCR was amplified with the following conditions: an initial denaturation at 94 °C for 2 min, followed by 35 cycles at 94 °C for 1 min, 52 °C for 1 min, and 72 °C for 1 min, with a final extension of 72 °C for 10 min. Multiplex PCRs were checked on a 3% agarose gel and all products were cleaned using ExoSAP-IT (Affymetrix, Inc., Santa Clara, CA, USA). All products were sequenced twice using the Big Dye Terminator v3.1 cycle sequencing kit (Applied Biosystems, Foster City, CA, USA). Sequencing was performed on an ABI 3730 DNA Analyzer (Applied Biosystems) using the M13 forward primer and the M13 reverse primer. All forward and reverse sequences were checked by eye and priming sites were trimmed using Geneious Pro v. 3.6.1 (https://www.geneious.com). Trimmed sequences were compared to BOLD (FISH-BOL) and BLAST (GenBank) databases to identify them to the lowest taxonomic category possible (e.g. genus and/or species). Fin trimmings were assigned a species level identification when (i) the closest matching subject sequence(s) from only one species exhibited either an exact match to the query or a maximum of 2 bp differences after a BLAST search and (ii) when BOLD also returned the same unambiguous species level identification. Fin samples that did not fulfill both criteria were only assigned to genus. There is also one global species complex (the ‘blacktip sharks’) that includes *Carcharhinus limbatus*, *C. leiodon*, *C. tilstoni*, and *C. amblyrhynchoides* that exhibit identical sequences for the smaller mini-barcoding fragments; all trimmings that exhibited these sequences were assigned to the blacktip complex. Identical sampling and identification protocols have been implemented fortnightly (February 2014–January 2015) or monthly (February 2015–present) in Hong Kong^7,9^. Therefore, we used trimmings collected and analyzed from the same sampling months in Guangzhou and Hong Kong for comparative species diversity analysis (see below; i.e. we used the same number of trimmings and sampling events per location, even though we had many more from Hong Kong).

A rarefaction curve was generated using iNEXT Online^15^ to estimate the total number of species (i.e., species diversity) at a given number of samples (i.e., abundance data) and sampling units (i.e., incidence data) for both locations, based on the unified rarefaction and extrapolation sampling curves of Hill numbers for q = 0, 1 and 2^16^. A sampling unit was defined as two randomly purchased bags of shark fin trimmings purchased from one randomly chosen vendor. Species diversity was estimated using only trimmings detected to the species or species complex level. Trimmings identified to the genus level or unidentified were not included in the diversity analyses. The number of bootstraps was set to 10,000 and the level confidence interval to 0.95. Abundance data were analyzed with an endpoint setting of 10,000 samples and incidence data with an endpoint setting of 500 sampling units. Based on the sampling protocol used, 500 sampling units would result in 10,000 trimmings. In addition, the total number of species in each market was estimated using SpadeR Online^17^ using abundance and incidence data with six different models (Table 1). The models use rare species frequencies to estimate the number of undetected species in each market with 95% confidence intervals.

**Table 1:** Species richness estimations for Guangzhou and Hong Kong using abundance and incidence data.

Model	Estimate	SE	95% CI
**(a) Guangzhou species richness based on abundance data**			
Homogeneous model (Chao & Lee 1992)	50.868	2.580	48.178–59.701
Chao1 (Chao, 1984)	54.557	5.955	48.935–76.508
Chao1-bc (Chao et al. 2005)	53.107	4.974	48.509–71.719
iChao1 (Chiu et al. 2014)	55.946	4.030	50.883–67.774
ACE^a^ (Chao & Lee, 1992)	57.427	6.313	50.487–78.178
ACE^a^-1 (Chao & Lee, 1992)	60.880	9.346	51.187–93.010
**(b) Guangzhou species richness based on incidence data**			
Homogeneous model (Chao & Lee 1992)	52.947	3.323	49.141–63.519
Chao2 (Chao, 1987)	68.116	14.384	53.296–117.818
Chao2-bc (Chao et al. 2005)	63.968	11.361	52.147–102.935
iChao2 (Chiu et al. 2014)	70.623	10.940	56.957–103.046
ICE^b^ (Lee & Chao, 1994)	63.123	8.564	53.068–89.835
ICE^b^-1 (Lee & Chao, 1994)	72.693	9.664	59.595–99.414
**(c) Hong Kong species richness based on abundance data**			
Homogeneous model (Chao & Lee 1992)	47.208	1.812	45.541–54.012
Chao1 (Chao, 1984)	58.490	12.451	47.896–107.835
Chao1-bc (Chao et al. 2005)	53.993	8.046	47.001–85.425
iChao1 (Chiu et al. 2014)	61.490	12.451	49.421–106.512
ACE^c^ (Chao & Lee, 1992)	52.023	5.060	46.978–69.943
ACE^c^-1 (Chao & Lee, 1992)	54.513	7.578	47.408–82.586
**(d) Hong Kong species richness based on incidence data**			
Homogeneous model (Chao & Lee 1992)	50.216	3.112	46.763–60.430
Chao2 (Chao, 1987)	55.457	7.601	47.918–82.478
Chao2-bc (Chao et al. 2005)	53.580	6.358	47.346–76.380
iChao2 (Chiu et al. 2014)	55.457	7.601	47.918–82.478
ICE^d^ (Lee & Chao, 1994)	57.044	6.916	49.230–79.289
ICE^d^-1 (Lee & Chao, 1994)	60.739	10.063	49.996–94.581

^a^Abundance-based coverage estimator (ACE).

^b^Incidence-based coverage estimator (ICE).

^c^Abundance-based coverage estimator (ACE).

^d^Incidence-based coverage estimator (ICE).

To estimate the species composition of the fin trimmings in Guangzhou markets, we used a Poisson multinomial model and a Bayesian framework with non-informative priors to estimate the parameters. For model details see Fields et al.^9^. The model was fitted using JAGS software^18^ through R (R2Jags package)^19^. Data that included species that made up > 20 trimmings were used to fit to the models. The Deviance Information Criterion (DIC) was used to determine the model that best predicted the species composition in our dataset. After the model was fitted to the species that made up > 20 trimmings, the cutoff was adjusted downward as long as the model would continue to converge. Species below the cutoff were grouped by genus (*Carcharhinus* [requiem sharks] and *Callorhinchus* [chimaeras]) or binned into an “Other” category, each of which were large enough to be modeled. We conservatively estimated the proportion of each species using the final model output, without making any assumptions about the unidentified trimmings (i.e., samples that could not be identified after multiple attempts), which were included in the model on their own category. Hong Kong trimmings were not modeled since both previous studies have modeled these exact same data using the same statistical model, allowing for a direct comparison of the composition and proportion of species in both markets.

## Results

A total of 2,000 shark fin trimmings were collected and analyzed from the Guangzhou shark fin market with a successful identification of 86.3% to the species or genus level. The remainder (13.7%) failed to amplify after multiple attempts. Successfully identified trimmings (N = 1,706) comprised 43 species or species complexes and another 10 categories identified only to the genus level (Table 2). Most of these species were sharks, but batoids (Family Rhinidae) and chimaeras (Family Callorhinchidae) were also present. Ten oceanic shark species were identified (23.2% of all species present) that comprised the majority (71.6%) of all trimmings identified to the species/species complex level (Table 2). All of the remaining species (83.7% of all species recorded, 28.4% of trimmings) were coastal. Many (41.5%) of the species and species groups identified are threatened with extinction based on the International Union for Conservation of Nature (IUCN), and species in these categories represented 39.2% of the identified trimmings (Table 2).

**Table 2:** Species or species groups in the Guangzhou shark fin market and an updated species list from the Hong Kong retail markets with conservation status of each species.

Order	Scientific name	Common name	IUCN	CITES status	Size	Habitat	CountGZ	% of samples GZ	Count HK	% of samples HK
Carcharhiniformes	*Prionace glauca*	Blue shark	NT		Large	Oceanic	616	36.11	2,979	39.01
Carcharhiniformes	*Carcharhinus falciformis*	Silky shark	VU	Appendix II	Large	Oceanic	327	19.17	973	12.74
Carcharhiniformes	*Carcharhinus* spp.	Requiem sharks					139	8.15	336	4.40
Lamniformes	*Isurus oxyrinchus*	Shortfin mako shark	EN		Large	Oceanic	71	4.16	181	2.37
Carcharhiniformes	*Sphyrna lewini*	Scalloped hammerhead shark	CR	Appendix II	Large	Oceanic	71	4.16	323	4.23
Carcharhiniformes	*Sphyrna zygaena*	Smooth hammerhead shark	VU	Appendix II	Large	Oceanic	62	3.63	276	3.61
Carcharhiniformes	*Carcharhinus limbatus, C. leiodon, C. tilstoni, C. amblyrhynchoides*	Blacktip, Graceful, Smoothtooth blacktip, Australian blacktip sharks	NT		Large	Coastal	37	2.17	356	4.66
Carcharhiniformes	*Rhizoprionodon acutus*	Milk shark	LC		Small	Coastal	31	1.82	158	2.07
Carcharhiniformes	*Mustelus punctulatus*	Blackspotted smooth-hound shark	DD		Small	Coastal	31	1.82	75	0.98
Carcharhiniformes	*Mustelus schmitti*	Narrownose smooth-hound shark	EN		Small	Coastal	29	1.70	57	0.75
Lamniformes	*Alopias pelagicus*	Pelagic thresher shark	EN	Appendix II	Large	Oceanic	28	1.64	122	1.60
Carcharhiniformes	*Carcharhinus longimanus*	Oceanic whitetip shark	CR	Appendix II	Large	Oceanic	27	1.58	63	0.83
Carcharhiniformes	*Carcharhinus sorrah*	Spot-tail shark	NT		Small	Coastal	26	1.58	74	0.97
Chimaeriformes	*Callorhinchus* spp.	Chimaeras			Small	Coastal	24	1.41	160	2.10
Carcharhiniformes	*Carcharhinus leucas*	Bull shark	NT		Large	Coastal	22	1.29	129	1.69
Carcharhiniformes	*Scoliodon* spp.				Small	Coastal	15	0.88	0	0.00
Carcharhiniformes	*Carcharhinus amboinensis*	Pigeye shark	DD		Large	Coastal	14	0.82	113	1.48
Carcharhiniformes	*Mustelus* spp.	Smooth-hound sharks			Small	Coastal	13	0.76	30	0.39
Carcharhiniformes	*Galeocerdo cuvier*	Tiger shark	NT		Large	Coastal	10	0.59	47	0.62
Carcharhiniformes	*Carcharhinus brevipinna*	Spinner shark	NT		Large	Coastal	10	0.59	102	1.34
Lamniformes	*Alopias superciliosus*	Bigeye thresher shark	VU	Appendix II	Large	Oceanic	9	0.53	7	0.09
Carcharhiniformes	*Rhizoprionodon* spp.	Sharpnose sharks			Small	Coastal	9	0.53	20	0.26
Lamniformes	*Lamna ditropis*	Salmon shark	LC		Large	Oceanic	7	0.41	78	1.02
Carcharhiniformes	*Carcharhinus altimus/plumbeus*	Bignose/Sandbar shark	DD/VU		Large	Coastal	6	0.35	51	0.67
Carcharhiniformes	*Carcharhinus albimarginatus*	Silvertip shark	VU		Large	Coastal	6	0.35	10	0.13
Carcharhiniformes	*Sphyrna mokarran*	Great hammerhead shark	CR	Appendix II	Large	Coastal	5	0.29	70	0.92
Carcharhiniformes	*Hemipristis elongata*	Snaggletooth shark	VU		Small	Coastal	5	0.29	14	0.18
Carcharhiniformes	*Hemigaleus australiensis*	Australian weasel shark	LC		Small	Coastal	4	0.23	3	0.04
Carcharhiniformes	*Mustelus mustelus*	Common smooth-hound shark	VU		Small	Coastal	4	0.23	12	0.16
Lamniformes	*Lamna nasus*	Porbeagle shark	VU	Appendix II	Large	Oceanic	4	0.23	9	0.12
Rhinopristiformes	*Rhynchobatus* spp.	Wedgefishes	CR		Large	Coastal	4	0.23	35	0.46
Squaliformes	*Squalus* spp.	Dogfishes			Small	Coastal	4	0.23	5	0.07
Carcharhiniformes	*Carcharhinidae*						3	0.18	0	0.00
Carcharhiniformes	*Galeorhinus galeus*	Soupfin shark	VU		Large	Coastal	3	0.18	24	0.31
Carcharhiniformes	*Carcharhinus melanopterus*	Blacktip reef shark	NT		Small	Coastal	3	0.18	7	0.09
Lamniformes	*Alopias* spp.	Thresher sharks	VU		Large	Oceanic	2	0.12	38	0.50
Carcharhiniformes	*Negaprion brevirostris*	Lemon shark	NT		Large	Coastal	2	0.12	5	0.07
Carcharhiniformes	*Carcharhinus obscurus/galapagensis*	Dusky/Galapagos shark	VU/NT		Large	Coastal	2	0.12	58	0.76
Carcharhiniformes	*Scoliodon macrorhynchos*	Pacific spadenose shark			Small	Coastal	2	0.12	0	0.00
Carcharhiniformes	*Mustelus canis*	Smooth dogfish	NT		Small	Coastal	2	0.12	68	0.89
Squaliformes	*Squalus acanthias*	Spiny dogfish	VU		Small	Coastal	2	0.12	12	0.16
Carcharhiniformes	*Rhizoprionodon porosus/terraenovae*	Caribbean/Atlantic sharpnose sharks	LC		Small	Coastal	2	0.12	21	0.28
Carcharhiniformes	*Carcharhinus acronotus*	Blacknose shark	NT		Small	Coastal	2	0.12	16	0.21
Carcharhiniformes	*Triaenodon obesus*	Whitetip reef shark	NT		Small	Coastal	2	0.12	1	0.01
Carcharhiniformes	*Carcharhinus brachyurus*	Bronze whaler shark	NT		Large	Coastal	2	0.12	16	0.21
Carcharhiniformes	*Rhizoprionodon taylori*	Australian sharpnose shark	LC		Small	Coastal	1	0.06	34	0.45
Carcharhiniformes	*Carcharhinus dussumieri*	Whitecheek shark	EN		Small	Coastal	1	0.06	21	0.28
Carcharhiniformes	*Carcharhinus porosus*	Smalltail shark	DD		Small	Coastal	1	0.06	4	0.05
Carcharhiniformes	*Mustelus henlei*	Brown smooth-hound shark	LC		Small	Coastal	1	0.06	16	0.21
Carcharhiniformes	*Lamiopsis* spp.	Broadfin sharks	EN		Small	Coastal	1	0.06	0	0.00
Carcharhiniformes	*Loxodon macrorhinus*	Sliteye shark	LC		Small	Coastal	1	0.06	2	0.03
Carcharhiniformes	*Carcharhinus amblyrhynchos*	Grey reef shark	NT		Large	Coastal	1	0.06	24	0.31
Carcharhiniformes	*Eusphyra blochii*	Winghead shark	EN		Large	Coastal	1	0.06	1	0.01
Squaliformes	*Dalatia licha*	Kitefin shark	NT		Large	Deep-benthic	0	0.00	72	0.94
Rhinopristiformes	*Rhynchobatus australiae*	White-spotted wedgefish	CR		Large	Coastal	0	0.00	45	0.59
Carcharhiniformes	*Negaprion acutidens*	Sicklefin lemon shark	VU		Large	Coastal	0	0.00	35	0.46
Squaliformes	*Centrophorus* spp.	Gulper sharks			Small	Deep-benthic	0	0.00	31	0.41
Orectolobiformes	*Chiloscyllium punctatum*	Bamboo shark	NT		Small	Coastal	0	0.00	22	0.29
Carcharhiniformes	*Carcharhinus limbatus*	Blacktip shark	NT		Large	Coastal	0	0.00	21	0.28
Orectolobiformes	*Chiloscyllium* spp.	Bamboo sharks			Small	Coastal	0	0.00	20	0.26
Rhinopristiformes	*Rhynchobatus laevis*	Smoothnose wedgefish	CR		Large	Coastal	0	0.00	17	0.22
Carcharhiniformes	*Mustelus mosis*	Arabian smooth-hound shark	DD		Small	Coastal	0	0.00	13	0.17
Carcharhiniformes	*Rhizoprionodon oligolinx*	Grey sharpnose shark	LC		Small	Coastal	0	0.00	11	0.14
Chimaeriformes	*Hydrogalus novaezealandiae*	Dark ghostshark	LC		Small	Deep-benthic	0	0.00	7	0.09
Carcharhiniformes	*Rhizoprionodon longurio*	Pacific sharpnose shark	DD		Small	Coastal	0	0.00	7	0.09
Carcharhiniformes	*Carcharhinus isodon*	Finetooth shark	LC		Small	Coastal	0	0.00	7	0.09
Chimaeriformes	*Hydrogalus* spp.	Other chimeras			Small	Deep-benthic	0	0.00	7	0.09
Orectolobiformes	*Chiloscyllium plagiosum*	Whitespotted bamboo shark	NT		Small	Coastal	0	0.00	6	0.08
Carcharhiniformes	*Carcharhinus macloti*	Hardnose shark	NT		Small	Coastal	0	0.00	6	0.08
Carcharhiniformes	*Carcharhinus amblyrhynchoides*	Graceful shark	NT		Large	Coastal	0	0.00	6	0.08
Squaliformes	*Centroscymnus coelolepis*	Portuguese dogfish	NT		Small	Deep-benthic	0	0.00	5	0.07
Carcharhiniformes	*Lamiopsis temminckii*	Broadfin shark	EN		Small	Coastal	0	0.00	5	0.07
Carcharhiniformes	*Glyphis* spp.	River sharks	EN		Large	Riverine/coastal	0	0.00	4	0.05
Squaliformes	*Deania profundorum*	Arrowhead dogfish	LC		Small	Deep-benthic	0	0.00	4	0.05
Lamniformes	*Isurus paucus*	Longfin mako shark	EN		Large	Oceanic	0	0.00	4	0.05
Carcharhiniformes	*Scoliodon laticaudus*	Spadenose shark	NT		Small	Coastal	0	0.00	4	0.05
Lamniformes	*Lamna* spp.				Large	Oceanic	0	0.00	3	0.04
Rhinopristiformes	*Rhynchobatus djiddensis*	Giant guitarfish	CR		Large	Coastal	0	0.00	3	0.04
Carcharhiniformes	*Mustelus lunulatus*	Sicklefin smooth-hound shark	LC		Small	Coastal	0	0.00	3	0.04
Carcharhiniformes	*Loxodon* spp.				Small	Coastal	0	0.00	3	0.04
Carcharhiniformes	*Sphyrna tiburo*	Bonnethead shark	LC		Small	Coastal	0	0.00	3	0.04
Carcharhiniformes	*Carcharhinus brevipinna/brachyurus*	Spinner/Bronze whaler shark			Large	Coastal	0	0.00	3	0.04
Carcharhiniformes	*Sphyrna* spp.	Hammerhead sharks					0	0.00	3	0.04
Squaliformes	*Centrophorus isodon*	Blackfin gulper shark	DD		Small	Deep-benthic	0	0.00	2	0.03
Lamniformes	*Alopias vulpinus*	Common thresher shark	VU	Appendix II	Large	Oceanic	0	0.00	2	0.03
Carcharhiniformes	*Mustelus californicus*	Grey smooth-hound shark	LC		Small	Coastal	0	0.00	2	0.03
Carcharhiniformes	*Sphyrna tudes*	Smalleye hammerhead shark	VU		Small	Coastal	0	0.00	2	0.03
Rhinopristiformes	*Glaucostegus* spp.	Giant guitarfishes	EN		Large	Coastal	0	0.00	1	0.01
Rhinopristiformes	*Pristis* spp.	Sawfishes	CR		Large	Coastal	0	0.00	1	0.01
Carcharhiniformes	*Hemigaleus microstoma*	Sicklefin weasel shark	VU		Small	Coastal	0	0.00	1	0.01
Chimaeriformes	*Callorhinchus callorynchus*	Elephantfish	LC		Small	Coastal	0	0.00	1	0.01
Lamniformes	*Carcharias taurus*	Sandtiger shark	VU		Large	Coastal	0	0.00	1	0.01
Hexanchiformes	*Hexanchus griseus*	Bluntnose sixgill shark	NT		Large	Coastal	0	0.00	1	0.01
Squatiniformes	*Squatina californica*	Pacific angel shark	NT		Small	Coastal	0	0.00	1	0.01
Orectolobiformes	*Stegostoma fasciatum*	Zebra shark	NT		Large	Coastal	0	0.00	1	0.01
Squaliformes	*Squalidae*						0	0.00	1	0.01
Rhinopristiformes	*Glaucostegus cemiculus*	Blackchin guitarfish	EN		Large	Coastal	0	0.00	1	0.01
Rhinopristiformes	*Rhyna ancylostoma*	Bowmouth guitarfish	EN		Large	Coastal	0	0.00	1	0.01
Squaliformes	*Deania* spp.	Deepwater dogfish sharks					0	0.00	1	0.01

The updated list for Hong Kong contains N = 7,636 successfully identified samples.

**Figure 1:**
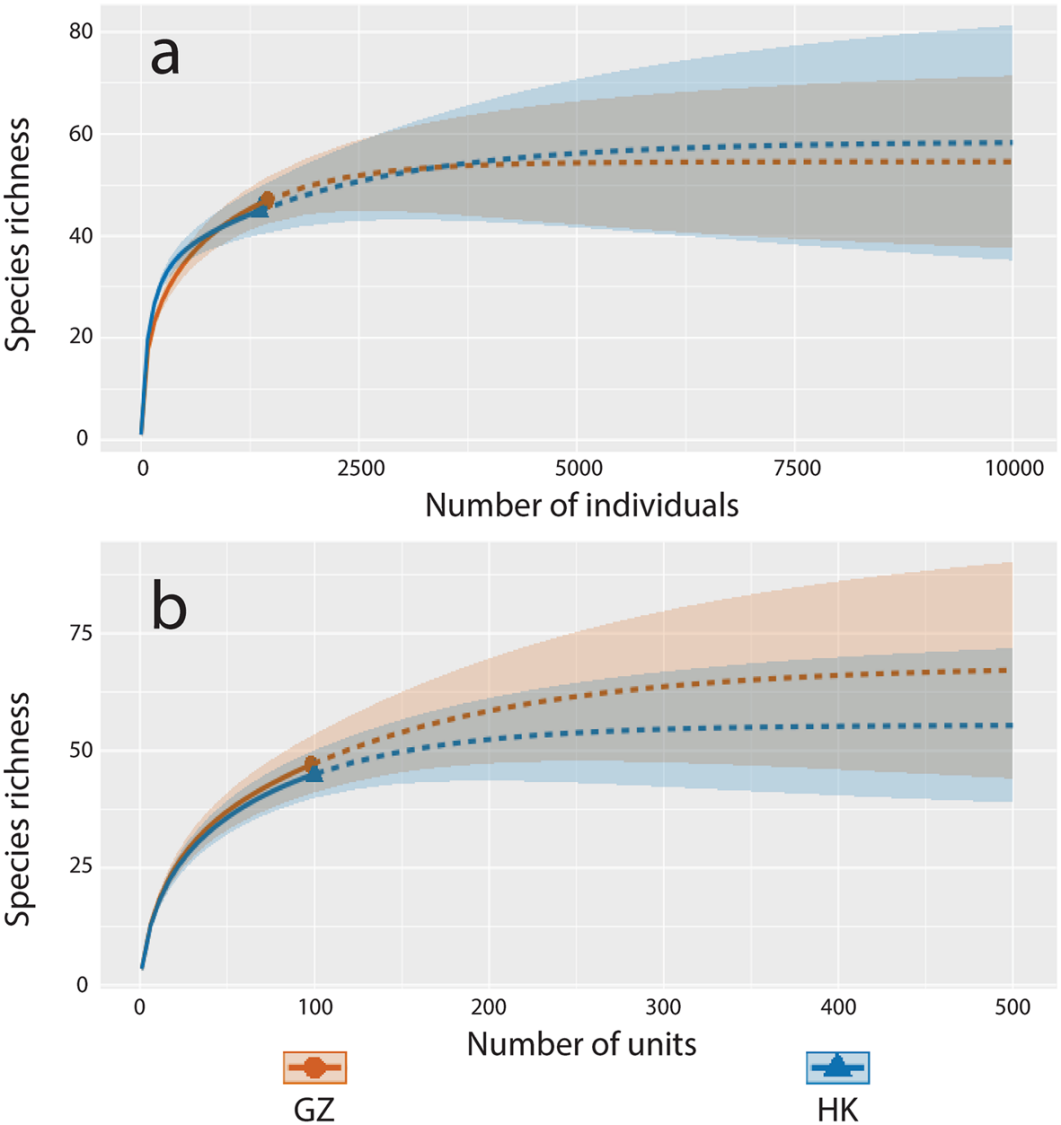
Species richness rarefaction curve for Guangzhou and Hong Kong using (**a**) abundance data and (**b**) incidence data.

**Figure 2:**
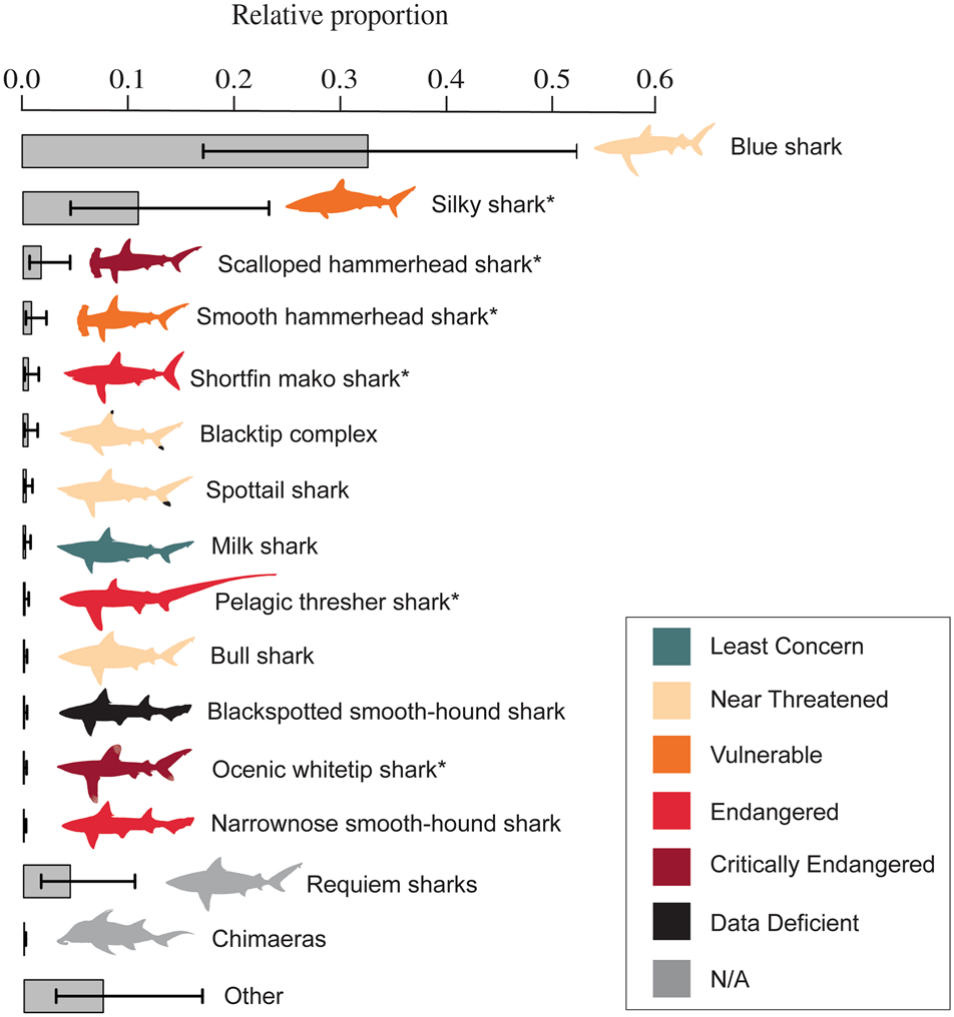
Bar-plot with 95% confidence intervals from Bayesian models showing the relative proportion of shark species, species complexes, and genera that made up > 20 trimmings. Blacktip complex denotes the species complex comprised of *Carcharhinus limbatus*,* C. amblyrhinchoides*,* C. leiodon*, and *C. tilstoni.* Species that made up < 20 trimmings for each of the three sampled years were binned by genus or grouped under “Other.” Frequency of unidentified samples is not shown.

The rarefaction curve for our sampling effort in Guangzhou and Hong Kong over the same period did not reach a plateau for runs with abundance and incidence data (Fig. 1). The extrapolation of the abundance data analysis reached a plateau at around 3,750 samples for both markets (Fig. 1a), while the extrapolation of the incidence data analysis reached it at around 300 sampling units (Fig. 1b). Extrapolation of the abundance and incidence data showed both markets being equally diverse (Fig. 1a,b). We determined that an additional maximum of 69 and 62 taxa occur in the Guangzhou and Hong Kong markets respectively, based on the minimum and maximum confidence intervals from the combined species richness estimates (Table 1). Modeling the species composition of the Guangzhou trimmings indicated that around 13 species comprised the vast majority, with the blue shark (*Prionace glauca*) the most common species overall (Fig. 2). CITES Appendix II listed silky (*Carcharhinus falciformis*), scalloped hammerhead (*Sphyrna lewini*), smooth hammerhead (*S. zygaena*), shortfin mako (*Isurus oxyrinchus*) threshers (genus *Alopias*; nearly all *A. pelagicus*), and oceanic whitetip (*C. longimanus*) modeled as the second, third, fourth, fifth, ninth and twelfth most common out of all species or species complexes identified (Fig. 2).

## Discussion

This study is the first assessment of the species composition of the largest shark fin market in Mainland China. The species identification methodology applied in this study to both Guangzhou and Hong Kong is a conservative approach that allows for the identification of nearly all elasmobranchs to the species level, with a few exceptions where species complexes are present^14^. The number of species found during our sampling of Guangzhou was 43 species or species complexes, excluding samples identified only to the genus level. This was very similar to the number found in Hong Kong with equivalent sampling effort (44). The total number of species found in Hong Kong (n = 81 species/species complexes) reported by Cardeñosa et al.^7^ over a longer sampling period (4 years) is similar to the prediction of the maximum confidence intervals from the species richness estimates and the abundance rarefaction curve from the smaller sampling effort in Guangzhou. Given this accuracy for Hong Kong, we suggest that a larger sampling effort in Guangzhou would yield a similar number of species overall.

While we present a robust comparative survey of species richness in these two markets our approach is conservative and therefore underestimates the total species richness. The statistical models we used to estimate total species richness from these data (i.e., Chao1, Chao1-bc, iChao1, ACE, and ACE1) use the frequencies of rare species in the sample to infer the number of undetected species and assume that species are not missed in the sample^15,16^. There are, however, a number of ways for us to miss species in our conservative species identification approach: the presence of species complexes that cannot be resolved with the genetic marker used (e.g., the blacktips), when the taxonomy of species in unresolved (e.g., within the ray family Rhinobatidae [wedgefish], where there are misidentified and misclassified sequences in GenBank and BOLD; and on occasions when our mini-barcodes are too short or contain nucleotide ambiguities that preclude identification lower than genus . Future work could sequence a longer portion of the COI or other loci for fin trimmings that could only be identified to genus or complex in the present study and use species delimitation approaches and/or comparisons to sequences from vouchered specimens to fully resolve the species richness of these markets.

The proportion of species or species groups in IUCN threatened categories was similar for the parallel sampling efforts: 37.9% (Hong Kong) and 41.8% (Guangzhou). The present study thus extends the previous work in Hong Kong^7,9^ by revealing that Guangzhou is trading fins from a similar diversity of sharks, rays, and chimaeras, and more than a third of the traded species exhibit high extinction risk. We also found that CITES-listed species were prevalent in Guangzhou, although potential latency of products imported prior to implementation in late 2014 (hammerheads, oceanic whitetips) or 2016 (silky, threshers) makes it difficult to pinpoint how much of this represents illicit trade (i.e., specimens imported into China without appropriate CITES documentation). It is also unclear how much of this originates from whole fins initially imported (and perhaps reported to CITES) into Hong Kong and then reexported and processed near Guangzhou.

Guangzhou exhibits a strong skew in species composition, being dominated by a small subset of the total species diversity (e.g., only 13 species represented by > 20 fin trimmings). Most of these were oceanic sharks that represented the largest proportions overall (71.6%)^7,9^. Skewed species composition was also characteristic of Hong Kong, with skews to many of the same species that dominated Guangzhou^7,9^. The only substantial difference in composition between the two sampling locations was that the shortfin mako was almost twice as common in the Guangzhou than in Hong Kong trimmings (i.e., 4.16% vs. 2.37%) and had a higher incidence (i.e., higher proportion of bags with identified shortfin mako shark trimmings). As a result, it modeled as the fifth most common species in Guangzhou where it was ninth behind several coastal species (e.g., spinner [*C. brevipinna*], bull [*C. leucas*], Java [*C. amboinensis*]) that were more common in Hong Kong^7^. This could potentially mean there are different and direct supply chains for this species into Mainland China, possibly their own high seas longline fleet, which may increase its presence in trimmings in Guangzhou relative to Hong Kong. This potential input into China is an important issue since the shortfin mako was recently listed on CITES Appendix II. The distant water fleet is now required to report landings of this species to CITES under “Introduction from the Sea” rules^8^.

The similarity between the species composition of fin trimmings in the shark fin markets of Hong Kong and Guangzhou extends previous studies that suggest these two markets are connected^10^ and is not surprising considering the proximity (129 km) and overland connections (road, rail) between these cities. Hong Kong has historically been the trading port of entry to mainland China, where fins arrive and are sent to the Guangdong Province for processing and processed fins are sent back to Hong Kong and other major cities in mainland China for consumption^10^. We suggest that some of the similarity we observed is driven by a similar supply chain for the trimmings: fins from Mainland China and Hong Kong are largely processed in Guangdong and resulting trimmings are then returned to these hubs for sale in their local retail markets. Although the border separating Hong Kong and Guangzhou is not international, CITES permits for listed species are required for transit (Hong Kong Agriculture Fisheries and Conservation Department [AFCD], pers comm). Given the prevalence of CITES listed species in both markets during our survey we suggest some surveillance investments for CITES listed shark products at the Hong Kong-China border is likely warranted^20,21^.

Despite the contemporary similarity found between the species composition of a fin market proxy in Hong Kong and Guangzhou, we suggest that capacity building and systematic studies of other fin trade hubs within Mainland China, Viet Nam, Singapore, Japan, Thailand, Taiwan, and Malaysia, are needed. Shark fin imports have declined sharply in both Hong Kong and especially China since 2011^2^ for a variety of potential reasons (e.g., changes in reporting and sourcing, new policies prohibiting extravagant spending by the governmental sector, reduced public demand), while imports have increased in some of these other hubs^2^. Since these hubs are less culturally and geographically connected to Hong Kong and Guangzhou, they are likely to have different inputs and preferences that could affect species composition. Indeed, some appear to focus on small, low value fins as opposed to the large valuable ones mainly traded in Hong Kong and China^2^. We therefore recommend investments in approaches to monitor the species composition of these hubs as well, in order to gain a clearer understanding of the species-specific dynamics of the international shark fin trade. Nonetheless, continued monitoring of the Hong Kong-Guangzhou hubs is necessary given the relatively high proportion of species threatened with extinction and/or listed under Appendix II of CITES in our surveys.

## Supplementary information

Supplementary information.

## Data Availability

The Guangzhou raw data used in this study is available as supplementary information. Further data on Hong Kong fin markets is available from the corresponding author upon request.
